# PLACE OF DEATH FOR PEOPLE WITH DEMENTIA: EVIDENCE FROM THE CDC WONDER MORTALITY DATA

**DOI:** 10.1093/geroni/igac059.1753

**Published:** 2022-12-20

**Authors:** Takashi Amano

**Affiliations:** Rutgers University-Newark, Newark, New Jersey, United States

## Abstract

Previous studies have found that patients with dementia experience poorer end-of-life care compared to patients with cancer. Dying in the preferred place has become a common measure of the quality of end-of-life care, and it has been consistently reported that the majority of people prefer to die at home. Thus, this study examines whether dying from dementia is a significant determinant of the place of death in mortalities among older adults. The Mortality Data on Center for Disease Control and Prevention (CDC) Wide-ranging Online Data for Epidemiologic Research (WONDER) between 2010 and 2019 were utilized. This study examined whether dying from dementia was associated with place of death (hospital, home, hospice facility, nursing home/long-term care facility) among deaths at 65 years or older. A multinomial logistic regression analysis was performed to estimate the association adjusted for covariates. We analyzed a total of 15,855,034 death records of which 12.34% were dementia deaths. The percentage of deaths at nursing homes was higher in deaths from dementia (56.5%) than other deaths (21.85%), whereas percentages of death at home were similar (20.7% for dementia death and 29.1% for other deaths). Multinomial logistic regression revealed that dementia deaths were 2.74 times more likely to occur at nursing homes than at hospitals compared to other deaths. Results suggest that dementia deaths are more likely to occur at nursing homes/long-term care facilities than other deaths. Further research should investigate the ways to improve the quality of end-of-life care for people with dementia in nursing homes/long-term care facilities.

